# Persulphate of Iron

**Published:** 1862-06

**Authors:** 


### PERSULPHATE OF IRON.

A WRITER in the Chicago Medical Journal details some experiments, and makes some suggestions, in regard to the use of persulphate of iron, in the treatment of polypi in the ear and nose, by its use he obtained good results.

Fearful of its influence upon the surrounding tissues, he adopted the following method of employment:

He prepares small cylinders of the salt, about a quarter of an inch in length, and scarcely an inch in diameter, from a thick paste of the iron and mucilage well rubbed together; he then punctures the tumor to a sufficient depth with a narrow knife, and then introduces one of these cylinders; this produces a coagulation of blood in the mass, and it is soon thrown off.

We would suggest trial of the persulphate in the treatment of spongy gums, or those of a prurient growth. May it not also be used with good results, in the treatment of alveolar abscess? Enlarge the canal to the sac, and introduce a small portion of the salt. Somebody please experiment and report. T.

